# Efficacy of Cipargamin (KAE609) in a Randomized, Phase II Dose-Escalation Study in Adults in Sub-Saharan Africa With Uncomplicated *Plasmodium falciparum* Malaria

**DOI:** 10.1093/cid/ciab716

**Published:** 2021-08-19

**Authors:** Esther K Schmitt, Gilles Ndayisaba, Adoke Yeka, Kwaku Poku Asante, Martin P Grobusch, Etienne Karita, Henry Mugerwa, Stephen Asiimwe, Abraham Oduro, Bakary Fofana, Seydou Doumbia, Guoqin Su, Katalin Csermak Renner, Vinay Kumar Venishetty, Sarfaraz Sayyed, Judith Straimer, Ivan Demin, Sarita Barsainya, Caroline Boulton, Preetam Gandhi

**Affiliations:** 1 Novartis Pharma AG, Basel, Switzerland; 2 Rinda Ubuzima, Kigali, Rwanda; 3 Infectious Diseases Research Collaboration, Busia, Uganda; 4 Kintampo Health Research Centre, Kintampo North Municipality, Ghana; 5 Centre de Recherches Médicales en Lambaréné, Lambaréné, Gabon; 6 Amsterdam University Medical Centers, Amsterdam, The Netherlands; 7 University of Tübingen, Tübingen, Germany; 8 Center for Family Health Research, Kigali, Rwanda; 9 Joint Clinical Research Centre, Kampala, Uganda; 10 Kabwohe Clinical Research Center and Mbarara University of Science and Technology, Mbarara, Uganda; 11 Navrongo Health Research Centre, Navrongo, Ghana; 12 Malaria Research and Training Center, Sotuba, Mali; 13 University Clinical Research Center, Bamako, Mali; 14 Novartis Pharmaceuticals Corporation, East Hanover, New Jersey, USA; 15 Novartis Institutes for BioMedical Research, Hyderabad, India; 16 Novartis Healthcare Pvt Ltd, Hyderabad, India; 17 Novartis Institutes for BioMedical Research, Emeryville, California, USA

**Keywords:** cipargamin, KAE609, falciparum malaria, sub-Saharan Africa, efficacy

## Abstract

**Background:**

Cipargamin (KAE609) is a potent antimalarial in a phase II trial. Here we report efficacy, pharmacokinetics, and resistance marker analysis across a range of cipargamin doses. These were secondary endpoints from a study primarily conducted to assess the hepatic safety of cipargamin (hepatic safety data are reported elsewhere).

**Methods:**

This phase II, multicenter, randomized, open-label, dose-escalation trial was conducted in sub-Saharan Africa in adults with uncomplicated *Plasmodium falciparum* malaria. Cipargamin monotherapy was given as single doses up to 150 mg or up to 50 mg once daily for 3 days, with artemether-lumefantrine as control. Key efficacy endpoints were parasite clearance time (PCT), and polymerase chain reaction (PCR)–corrected and uncorrected adequate clinical and parasitological response (ACPR) at 14 and 28 days. Pharmacokinetics and molecular markers of drug resistance were also assessed.

**Results:**

All single or multiple cipargamin doses ≥50 mg were associated with rapid parasite clearance, with median PCT of 8 hours versus 24 hours for artemether-lumefantrine. PCR-corrected ACPR at 14 and 28 days was >75% and 65%, respectively, for each cipargamin dose. A treatment-emerging mutation in the *Pfatp4* gene, G358S, was detected in 65% of treatment failures. Pharmacokinetic parameters were consistent with previous data, and approximately dose proportional.

**Conclusions:**

Cipargamin, at single doses of 50 to 150 mg, was associated with very rapid parasite clearance, PCR-corrected ACPR at 28 days of >65% in adults with uncomplicated *P. falciparum* malaria, and recrudescent parasites frequently harbored a treatment-emerging mutation. Cipargamin will be further developed with a suitable combination partner.

**Clinical Trials Registration:**

ClinicalTrials.gov (NCT03334747).

## Teaser

Cipargamin, at single doses of 50–150 mg, was associated with rapid parasite clearance (parasite clearance time ~8 hours) and PCR-corrected adequate clinical and parasitological response at day 29 of ≥65% in adult patients with uncomplicated *Plasmodium falciparum* malaria.

Cipargamin (KAE609/NITD609) is a novel spiroindolone antimalarial [1]. In a phase II trial in Thailand [2], cipargamin 30 mg/day for 3 days had a median parasite clearance time (PCT) of 12 hours for both *Plasmodium falciparum* and *Plasmodium vivax*. This compares favorably with artemisinin-based therapies such as artemether-lumefantrine (Coartem/Riamet^;^ Novartis; median PCT from 24 to 44 hours) [3]. Parasite clearance was not affected by kelch13 mutations associated with artemisinin resistance in *P. falciparum* [4]. Polymerase chain reaction (PCR)–corrected cure rates between 14% and 60% at day 28 were reported in a phase II trial in Vietnam for single doses between 10 mg and 30 mg with a trend for increased efficacy with increased doses [5]. The elimination half-life of approximately 23 hours supports once-daily dosing and there was no food effect observed [2, 6].

Cipargamin is active against all intraerythrocytic stages of *P. falciparum* [7] and gametocytes [8]. Spiroindolones disrupt sodium homeostasis in *Plasmodium* by inhibiting the Na+ transporting plasma membrane ATPase Plasmodium falciparum ATPase 4 (*PfATP4*) [1, 9]. Mutations in the *PfATP4* gene have been generated in vitro under prolonged drug pressure, leading to decreased cipargamin susceptibility [7, 10, 11].

Transient liver function test elevations were observed in some trial participants [1, 12, 13], and the current trial was designed primarily to assess the hepatic safety of cipargamin in an active-controlled setting by comparison to current standard of care (artemether-lumefantrine). Hepatic safety results will be described elsewhere. Here we describe the efficacy and pharmacokinetics of cipargamin, and analysis of cipargamin resistance markers, which were assessed as secondary objectives.

## METHODS

### Study Design and Setting

This was a multicenter, randomized, open-label, dose-escalation phase II trial, conducted in Mali, Gabon, Ghana, Uganda, and Rwanda. The protocol and all amendments were reviewed by the Independent Ethics Committee or Institutional Review Board for each center. The trial was conducted according to International Conference on Harmonisation (ICH) E6 Guidelines for Good Clinical Practice. The trial is registered with ClinicalTrials.gov (NCT03334747).

### Participants

Eligible patients were adults (≥18 years old and ≥45 kg body weight) with microscopic confirmation of acute uncomplicated *P. falciparum* malaria (parasitemia of 500 to 50 000/μL with axillary temperature ≥37.5ºC or oral/tympanic/rectal temperature ≥38.0ºC or history of fever during the previous 24 hours). Exclusion criteria included mixed *Plasmodium* infections and severe malaria according to World Health Organization (WHO) criteria [14]. Full exclusion criteria are provided in the Supplementary Appendix.

### Randomization and Dose Escalation

**Figure 1.**
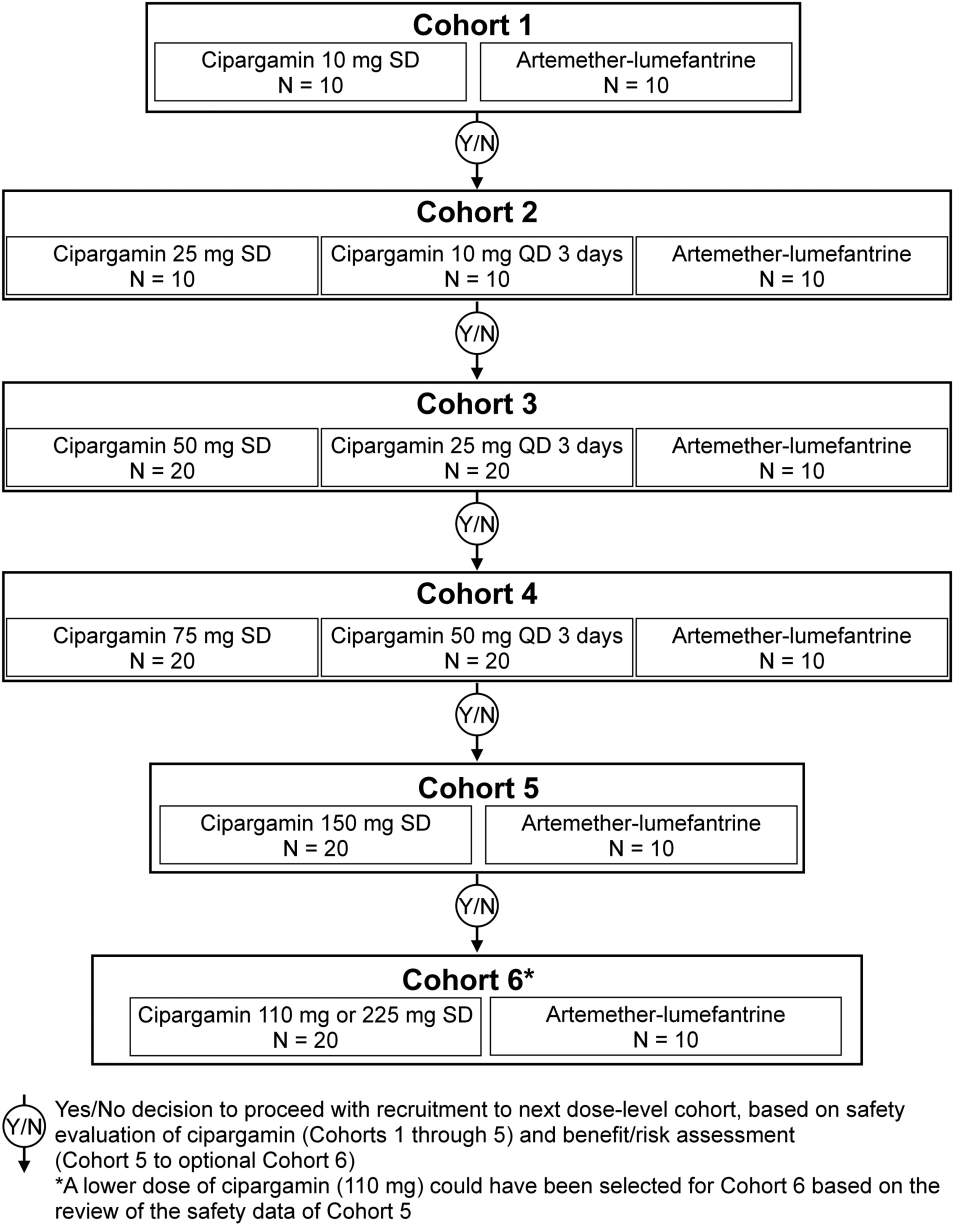
Study design. Abbreviations: QD, daily; SD, single dose.

Patients were treated in 5 cohorts, using ascending single or multiple doses of cipargamin (Figure 1). It was originally planned to enroll 4 cohorts, with maximum cipargamin dosages of a 75-mg single dose and 50 mg once daily for 3 days. Cohort 5 (150-mg single cipargamin dose) and an optional sixth cohort in which patients would receive 110-mg or 225-mg single doses, depending on hepatic safety in cohort 5, were added in a protocol amendment made when the trial was ongoing. If no added benefit was expected from using the 225-mg dose, the trial could be stopped after cohort 5. Cipargamin doses in the first 2 cohorts were potentially subtherapeutic, so the minimum number (N = 10) of patients needed to assess the primary objective was included. Subsequent cohorts each aimed to recruit 20 cipargamin patients. It was planned to treat 10 patients per cohort with artemether-lumefantrine (80/480 mg, twice daily for 3 days; Novartis Pharma AG) as an active comparator.

Recruitment to cohorts was sequential. After completion of each cohort, dose escalation of patients to the next cohort was determined by decision criteria (provided in Supplementary Table 1) based on postbaseline changes in Liver Function Test (LFTs) and approval by the safety review committee (SRC). The SRC (which included 1 external hepatologist) met formally after cohorts 1, 3, 4, and 5 had completed treatment.

Within each cohort, patients were randomized in parallel to treatment groups using Interactive Response Technology (IRT). After being contacted by the investigator, the IRT assigned a randomization number (using a validated system that automated the random assignment of patient numbers to randomization numbers) that linked the patient to a treatment arm and specified a unique medication number for the study drug to be dispensed to the patient. The randomization number was not communicated to the investigator. Randomization to a cohort could be suspended if patients experienced safety events.

Patients received close monitoring in an inpatient setting for at least the first 3 days, followed by frequent outpatient monitoring for a total of 4 weeks. Patients were required to yield 2 consecutive negative blood smears for parasites and clearance of fever to be discharged. Cipargamin patients who met protocol-specified treatment failure criteria received artemether-lumefantrine as rescue medication.

### Procedures

Blood samples were taken for parasite counts (Giemsa-stained thick and thin films) at baseline; at 2, 4, 8, 12, 24, 36, 48, 60, and 72 hours; then at days 4, 7, 10, 14, 21, and 28 after starting treatment, and at unscheduled visits. At least 200-thick film visual fields were examined. Parasite counts were made per 200 leukocytes (or if the count was <100 parasites, counting was continued for up to 500 leukocytes). The sampling schedule and method for pharmacokinetic analysis are provided in Supplementary Appendix. Parent drug concentrations in plasma samples were determined using a validated high-performance liquid chromatography–tandem mass spectrometry with electrospray ionization, with a lower limit of quantification of 1 ng/mL for cipargamin [5, 6]. PCR genotyping to assess recrudescence versus reinfection and identify resistance markers [15, 16] was performed at baseline and at the time of treatment failure. Methods for *Pfatp4* analysis are provided in the Supplementary Appendix.

### Outcomes and Measurements

Planned efficacy outcomes were as follows: PCR-corrected and uncorrected adequate clinical and parasitological response (ACPR) at 14 and 28 postdose; PCT; fever clearance time (FCT); proportions of patients with parasitemia at 12, 24, and 48 hours; parasite reduction ratio at 24 hours (PRR24); incidence of reinfection and recrudescence at 28 days; incidence of early treatment failure (ETF; defined in this study [Supplementary Table 2] more strictly than in the WHO definition [17] as subtherapeutic doses of cipargamin were used in the first 2 cohorts); late clinical failure (LCF); and late parasitological failure (LPF).

Pharmacokinetic parameters calculated were maximum plasma concentration (C_max_); time to maximum plasma concentration (T_max_); areas under the concentration-time curve from time zero to the time of the last quantifiable concentration (AUC_last_), zero to infinity (AUC_inf_), and zero to 24 hours (AUC_0-24h_); and elimination half-life (T_1/2_).

### Statistical Analysis

Analyses of efficacy variables were based on the full analysis set (all randomized patients who took at least 1 dose of study treatment during the treatment period and whose baseline parasite count was greater than zero). Artemether-lumefantrine groups from all cohorts were pooled for analysis. The ACPR was calculated for each treatment by cohort, with 95% confidence intervals (CIs) provided using the Clopper-Pearson method, as were rates of ETF, LCF and LPF, and proportions of patients with parasitemia by time point. For PCT and FCT, descriptive statistics (mean, standard error, median, quartiles) were presented for each treatment by cohort using the Kaplan-Meier method. Incidence rates of recrudescence and reinfection at day 29 were estimated using the Kaplan-Meier method based on the subset of full analysis set patients with clearance of initial infection before day 15. For PRR24 hours, descriptive statistics and 95% CI for the geometric mean were provided by cohort and treatment. If the asexual parasite count at hour 24 was 0, the half value of detection limit was used to calculate the ratio. The detection limit was assumed to be 20 parasites/µL. Pharmacokinetic analyses were based on the pharmacokinetic analysis set (all patients in the safety analysis set who had evaluable pharmacokinetic parameter data and took at least 80% of the assigned study medication). Noncompartmental analysis was used and descriptive statistics were provided. Two-sided 90% CIs were only calculated for AUC_0-24h_, C_max_, and T_max_.

## RESULTS

### Study Patients

The trial started on 16 November 2017 and was completed on 23 November 2019. A total of 188 patients (11 in Mali, 16 in Gabon, 29 in Ghana, 58 in Uganda, and 74 in Rwanda) were randomized in 5 cohorts (Supplementary Figure 1), 137 to cipargamin and 51 to artemether-lumefantrine. Two cipargamin patients were randomized but not treated and were excluded from the efficacy and pharmacokinetic analyses, and a further 2 patients were excluded from the pharmacokinetic analyses.

Patient demographics were comparable across treatment groups and consistent with the intended target population (Supplementary Table 3). Cohorts were balanced in terms of baseline characteristics, except for *P. falciparum* count, which tended to be higher in cohorts 4 and 5 (median asexual forms/µL in cipargamin groups between 8190 and 15 697) than cohorts 1 to 3 (850 to 6430). Cohorts 1 and 2 used potentially subtherapeutic doses: in view of this, the range of parasitemia permitted for inclusion in the study was low (500 to 50 000 parasites/µL).

### Efficacy

**Figure 2.**
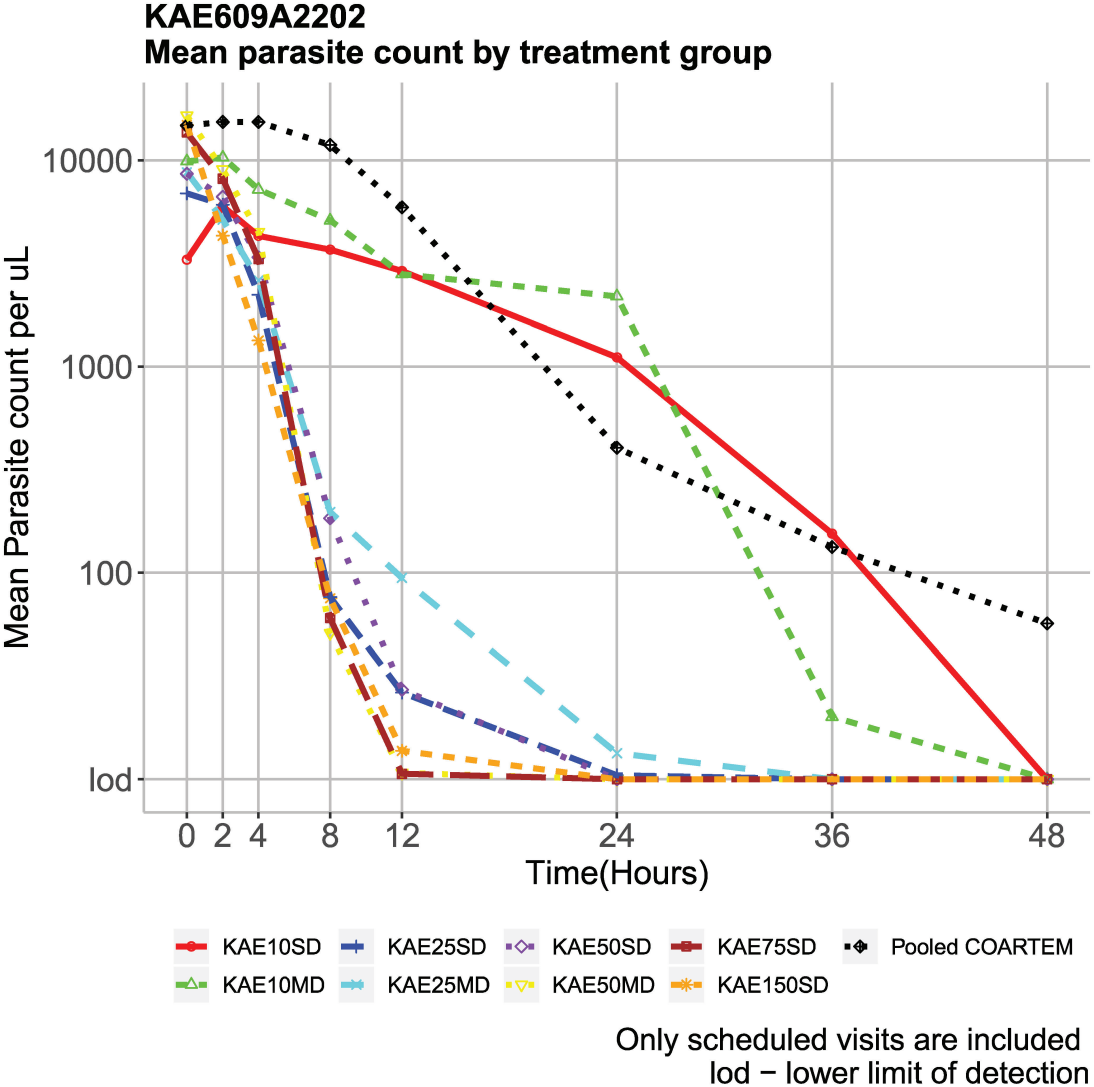
Mean parasite count over time (at scheduled visits only) by treatment group.

**Figure 3.**
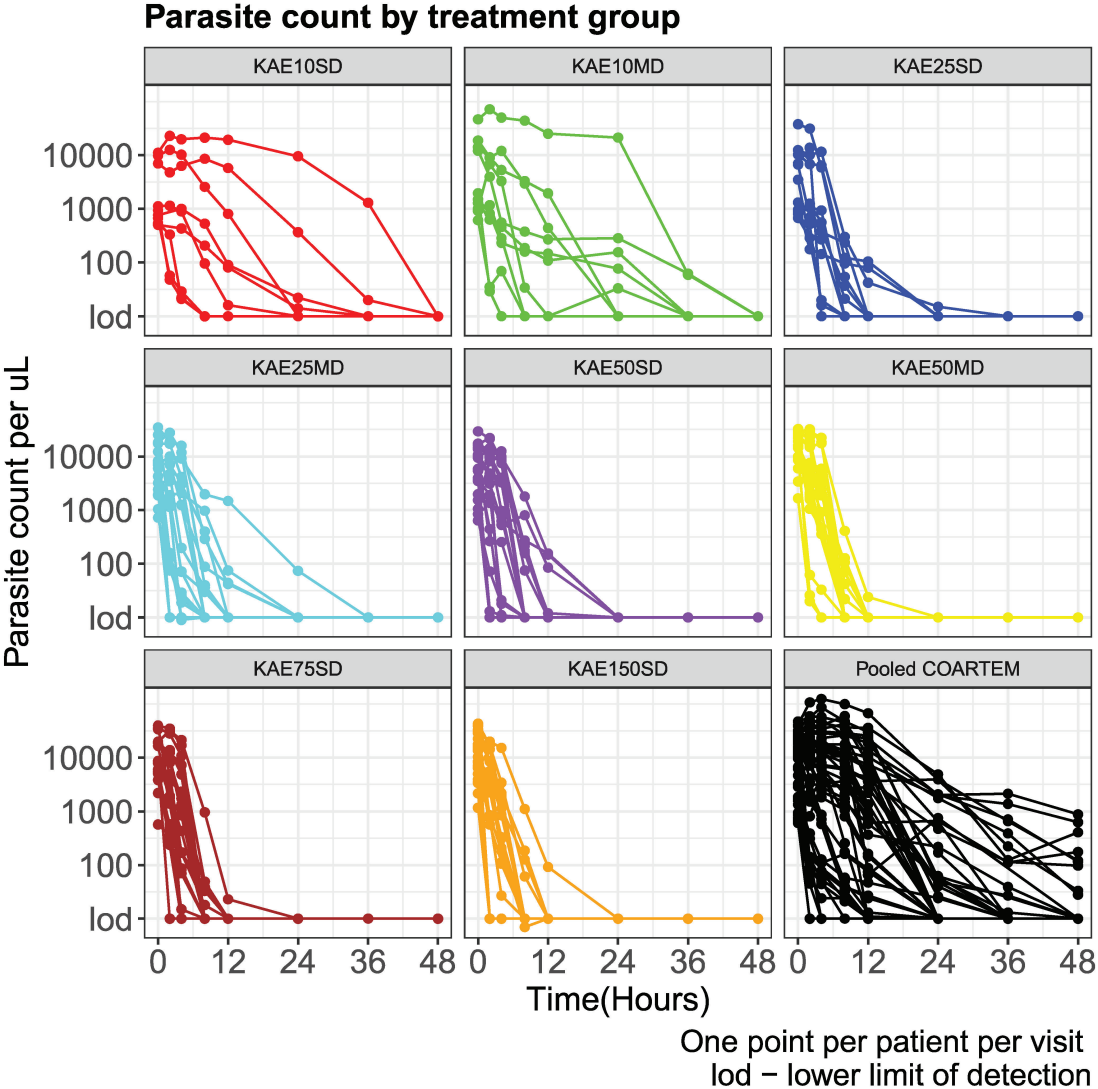
Individual patient parasite counts over time by treatment group; 1 point per patient per visit.

All cipargamin doses 50 mg or higher were associated with very rapid parasite clearance, with a median PCT of 8 hours compared with 24 hours for artemether-lumefantrine, with an apparent dose response plateauing at the 50-mg single dose (Table 1). Figure 2 shows mean parasitemia by treatment over time, and the observed dose response is consistent with that for PCT. Parasite clearance profiles by dose for individual patients are presented in Figure 3 and show very rapid clearance of parasitemia at cipargamin doses of 50 mg or higher.

**Table 1.** Parasite Clearance Time (Hours) by Treatment Group

Cipargamin Dose/Regimen									
		10 mg QD		25 mg QD		50 mg QD			
	10 mg SD (n = 10)	3 days (n = 10)	25 mg SD (n = 12)	3 days (n = 20)	50 mg SD (n = 21)	3 days (n = 19)	75 mg SD (n = 21)	150 mg SD (n = 22)	Artemether-lumefantrine (n = 51)
Median PCT	24.4	30.1	11.6	8.1	8.2	8.2	8.0	8.1	24.3
(2-sided 95% CI)	(8.0, 48.0)	(4.2, 36.7)	(8.0, 24.0)	(8.0, 12.2)	(8.0, 12.2)	(8.0, 12.0)	(8.0, 8.1)	(2.1, 9.2)	(24.1, 36.0)

PCT was calculated from the date of first treatment and is based on uncorrected parasite counts. Patients without parasite clearance for whatever reason are censored at the time of last parasite assessment. In the case that a patient receives rescue medication before parasite clearance, the time to event is censored at the first use of rescue medication.

Abbreviations: CI, confidence interval; PCT, parasite clearance time; QD, daily; SD, single dose.

Parasite reduction ratios at 24 hours increased with cipargamin dose. Peak median PRR24 around 1000 was achieved with the 50-mg single dose, whereas median PRR24 for the 25-mg single dose was approximately 600 and was below 100 for the 10-mg single dose. Due to the rapid parasite clearance with higher cipargamin doses, such that postbaseline parasite levels were undetectable in many patients, and the exclusion of patients with more than 50 000 parasites/µL from the study, the observed PRR24 for those doses may not reflect the actual values possible.

The FCT could not be meaningfully assessed in cohorts 1 to 3 due to the small numbers of patients with pyrexia, which was probably related to antipyretic treatment with paracetamol (acetaminophen). In cohorts 4 and 5, FCT was shorter with all cipargamin dose regimens (means ranging from 5.7 to 9.9 hours) than the pooled artemether-lumefantrine group (13.2 hours).

**Figure 4.**
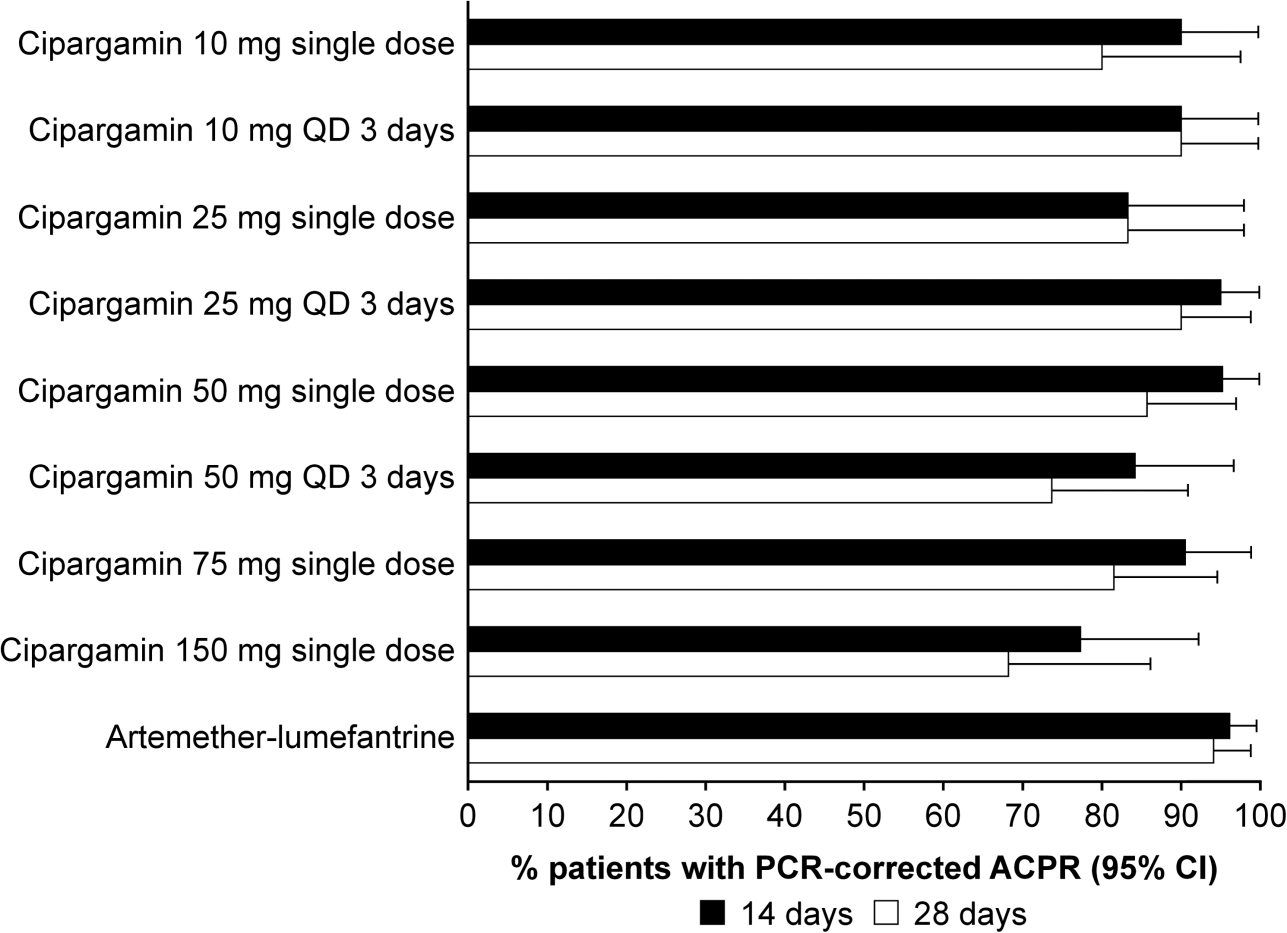
PCR-corrected ACPR by treatment group. Abbreviations: ACPR, adequate clinical and parasitological response; CI, confidence interval; PCR, polymerase chain reaction; QD, daily.

The PCR-corrected ACPRs at 14 and 28 days of over 75% and 65%, respectively (Figure 4), were achieved in all single- and multiple-dose arms. The PCR-uncorrected ACPR (Supplementary Table 4) in most cipargamin dose groups was lower than the PCR-corrected ACPR, especially at 28 days, reflecting reinfection rates. The recrudescence rate at 28 days was 24/135 (17.8%) for cipargamin; the Kaplan-Meier probability of recrudescence across cipargamin groups ranged from 10% to 32.5%. There was no obvious relationship of ACPR, rates of reinfection, or recrudescence to cipargamin dose. Differences between cohorts in baseline parasite counts (medians ranging from 850/µL in the cipargamin 10-mg single-dose group to 15 697/µL in the 50-mg/day for 3 days group), and the sequential recruitment by cohort leading to additional seasonal and geographic variability, complicate comparisons between cohorts. As expected, patients receiving artemether-lumefantrine had lower rates of reinfection and recrudescence. Early treatment failure (according to the study definition) only occurred in 1 artemether-lumefantrine patient; LCF and LPF were more common in cipargamin-treated patients (Supplementary Table 2).

### Resistance Marker Analysis

Baseline samples did not show any known mutations associated with resistance in the *Pfatp4* gene. One specific mutation in the *Pfatp4* gene, G358S, was detected in samples from 22 of 34 patients treated with cipargamin at the time of treatment failure (Table 2). A full list of identified mutations is available in Supplementary Table 6. The G358S mutant appeared to be more common in recrudescences with higher doses of cipargamin, which may reflect greater selective pressure and/or higher parasite counts in cohorts receiving such doses. All 34 patients with treatment failure after cipargamin monotherapy were successfully treated with standard of care (artemether-lumefantrine), including recrudescences with the mutant (G358S) parasites. The PCT in cipargamin-treated patients infected with parasites with kelch13 mutations at baseline did not differ significantly from patients with wild-type kelch13. Details on the observed prevalence of kelch13 mutations are reported separately [18].

**Table 2.** Occurrence of Recrudescence With *PfATP4* G358S Mutation, by Dose Regimen

		Patients, n (%)	
Dose regimen	Number of Patients	Late Treatment Failures	Recrudescences With G358S Mutation
Cipargamin 10 mg SD	9	1 (11)	0 (–)
Cipargamin 25 mg SD	12	4 (33)	0 (–)
Cipargamin 50 mg SD	21	4 (19)	4 (19)
Cipargamin 75 mg SD	20	5 (25)	3 (15)
Cipargamin 150 mg SD	22	9 (41)	5 (23)
Cipargamin 10 mg QD 3 days	10	1 (10)	1 (10)
Cipargamin 25 mg QD 3 days	20	4 (20)	3 (15)
Cipargamin 50 mg QD 3 days	19	6 (32)	6 (32)
Total	133	34 (26)	22 (17)

Abbreviations: QD, daily; *PfATP4*, Plasmodium falciparum ATPase 4; SD, single dose.

### Pharmacokinetics

Summary statistics for pharmacokinetic parameters are presented in Supplementary Table 5. After a single dose, median T_max_ of cipargamin in plasma ranged from 4 to 8 hours; mean C_max_, AUC_last_, and AUC_0-24h_ increased with dose over the tested range (10 to 150 mg) and were approximately dose-proportional. Pharmacokinetic parameters after multiple doses were consistent with those after single doses. High variability in exposure (coefficient of variation: 25–53%) was observed across cohorts similarly to previous studies [2] and might be due to variability in ɑ1-acid glycoprotein levels in patients [1]. Mean elimination half-life ranged from 24.4 to 35.1 hours after a single dose and 29.9 to 32.4 hours after 3 days of once-daily dosing; steady state was not attained after 3 days. The mean accumulation ratio was 1.4 following once-daily doses for 3 days.

**Figure 5.**
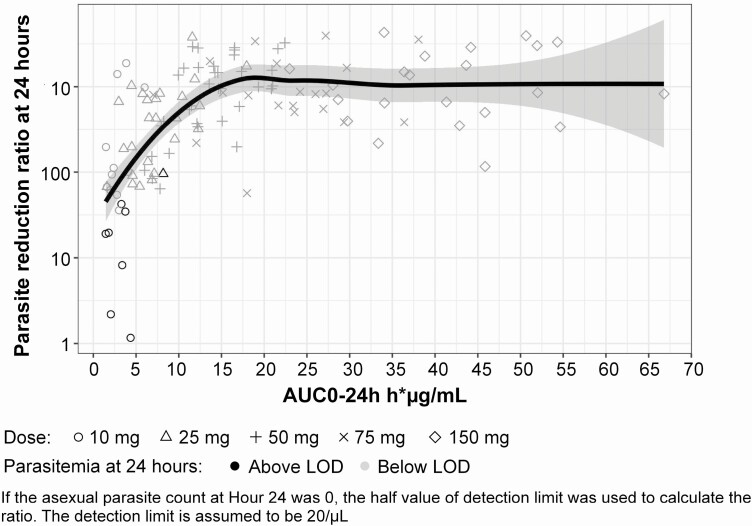
Exposure–response scatterplot for PRR24 and AUC0-24h for cipargamin-treated patients. Black symbols represent data with parasitemia counts at 24 hours above the limit of detection; gray symbols represent data with parasitemia counts at 24 hours below the limit of detection; black solid line: local regression line; gray-shaded area: confidence interval around the local regression line. Abbreviations: AUC0-24h, area under the concentration-time curve from zero to 24 hours; LOD, limit of detection; PRR24, parasite reduction ratio at 24 hours.

The exposure–response relationship for PRR24 was explored. PRR24 increased steeply with cipargamin exposure. Peak PRR24 appeared to be at an exposure of 15–20 μg.hr/mL and remained stable at higher exposures (Figure 5). For patients with exposures of approximately 10 μg.hr/mL or greater, there was no detectable parasitemia 24 hours postdose. This may have complicated the interpretation at higher exposures, where the curve may not reflect the true relationship between PRR24 and exposure. Dose selection for future studies could target an exposure above 15 μg.hr/mL for achieving peak PRR24, as in patients with malaria in this study, exposures as high as approximately 50 μg.hr/mL were observed without safety concerns.

## DISCUSSION

In recent years, mortality from malaria has declined, mainly due to the deployment of vector control measures and antimalarials based on artemisinin derivatives [14]. Artemisinin-based combination therapies provide rapid clearance of parasitemia and high ACPR. The emergence of resistance in South-East Asia [19-21], and more recently in Rwanda [22], threatens the utility of artemisinins, might derail current malaria control efforts, and highlights the need to develop novel antimalarials.

Cipargamin is a novel spiroindolone antimalarial that has shown rapid parasite clearance in phase II studies [1, 2, 5]. The current trial was conducted to assess the hepatic safety of cipargamin, following the observation of transient, mainly asymptomatic, LFT elevations in previous studies. Here we describe the efficacy and pharmacokinetics of cipargamin from this study, across a wide range of doses (10-mg to 150-mg single dose and 10-mg to 50-mg daily for 3 days). This was the first clinical trial to be performed with cipargamin in sub-Saharan Africa.

This study was not powered to assess efficacy, and the sequential cohorts and differences in baseline parasite counts complicate comparisons of efficacy between treatment arms. Patients were adults in sub-Saharan Africa (who are likely to have acquired partial immunity against malaria), so efficacy results may not be easily generalizable to other age groups or regions [2, 5].

In previous studies, cipargamin treatment was notable for the rapid clearance of parasites [2, 5]. Results from this study support this observation and show a clear dose–response relationship, demonstrated by PCT, PRR, and the time course of parasitemia. Artemisinin is regarded as a fast-acting antimalarial, and it is notable that cipargamin at doses of 50 mg and above showed considerably shorter PCT than artemether-lumefantrine (median values of ~8 hours and 24 hours, respectively). This was reflected in the time course of parasitemia reduction.

The ACPR, reported here across a wide cipargamin dose range, including 5 single-dose arms, was similar across treatment groups at day 15, but at day 29, recrudescence, LCF, and LPF were more frequent with cipargamin than with artemether-lumefantrine. This might be expected as cipargamin was used as monotherapy and the elimination half-life is approximately 24 hours. Other antimalarials with similar or shorter half-lives have also shown high rates of recrudescence when used as monotherapies [23, 24]. In an early study in China, artemether monotherapy with 4 doses (twice daily for 2 days) showed an uncorrected ACPR of 46% [3]. Cipargamin will be developed as the fast-acting component in a combination regimen and efficacy of the combination needs to be noninferior in all age groups compared with standard of care.

As with other PfATP4 inhibitors, mutations in the *PfATP4* gene that lead to decreased susceptibility have been selected in vitro under prolonged drug pressure of cipargamin, which indicates the polymorphic nature of the gene [7, 10, 11, 25]. In cases of treatment failure with cipargamin, a specific *PfATP4* mutation (G358S) was common. The G358S mutant was first described from in vitro experiments and was generated under selection pressure for SJ733 [26], a dihydroisoquinolone antimalarial candidate compound that targets PfATP4 [27]. Since this and other known *PfATP4* mutations were not detected in any of the baseline samples, this suggests that there is no pre-existing cipargamin resistance. Treatment with cipargamin monotherapy appears to have selected resistant parasites that arise spontaneously and are likely to be present in small numbers in patients with high parasitemia and undetectable at baseline [25]. This observation is consistent with preclinical data for cipargamin demonstrating a medium risk for resistance [7]. All patients with recrudescent parasites, including those with the G358S mutation, were successfully treated with artemether-lumefantrine. This confirms previous in vitro and clinical data regarding lack of cross-resistance between artemisinins and cipargamin and distinct modes of action [2, 7]. Further in vitro characterization of the G358S mutant is needed to better understand potential fitness costs and transmissibility. In future clinical studies, monitoring of resistance markers will be advised, and selection for resistance should be prevented by combining cipargamin with a long-acting partner compound with a high barrier to resistance.

In conclusion, cipargamin, at single doses of 50 mg to 150 mg, was associated with very rapid parasite clearance and PCR-corrected ACPR at day 29 of at least 65% in adult patients with uncomplicated *P. falciparum* malaria. This monotherapy study confirms the need for fixed-dose combination therapy to avoid recrudescence and selection for resistant parasites. Cipargamin, with its high potency, rapid parasite clearance, and potential for single-dose cure will be further developed for uncomplicated malaria with a suitable combination partner.

## Supplementary Data

Supplementary materials are available at *Clinical Infectious Diseases* online. Consisting of data provided by the authors to benefit the reader, the posted materials are not copyedited and are the sole responsibility of the authors, so questions or comments should be addressed to the corresponding author.

ciab716_suppl_Supplementary_Materials

## Notes

***Acknowledgments.*** The authors thank the patients who participated in this trial, all the investigators and trial site management and staff, and local community leaders. They also thank Phil Hunt of Southdown Medical Writing Ltd for assistance with preparation of the manuscript.

***Financial support.*** The sponsor of the study, Novartis Pharma AG, received financial support from Wellcome Trust (grant number 207813/Z/17/Z).

***Potential conflicts of interest.*** E. K. S., G. S., K. C. R., V. K. V., S. S., J. S., I. D., S. B., C. B., and P. G. are employed by and shareholders of Novartis. G. N., A. Y., K. P. A., M. P. G., E. K., H. M., S. A., A. O., B. J., and S. D. are principal investigators at the clinical sites for the study. M. P. G. reports nonpersonal consultancy fee for malaria expert advice (not related to the work presented here) by Novartis paid to his employer Amsterdam University Medical Centers (AMC). K. P. A. reports personal fees from Kintampo Health Research Centre for employment. E. K. reports employment from the Center for Family Health Research. H. M. reports personal fees from Novartis Pharma AG and personal fees for employment from the Joint Clinical Research Centre. G. F. N. reports personal fees from Rinda Ubuzima. A. O. reports personal fees for employment from the Navrongo Health Research Centre. S. A. reports personal fees for study time effort compensation from the Kabwohe Clinical Research Center KCRC outside the submitted work. A. Y. reports personal fees for employment from Infectious Diseases Research Collaboration and Makerere University. All authors have submitted the ICMJE Form for Disclosure of Potential Conflicts of Interest. Conflicts that the editors consider relevant to the content of the manuscript have been disclosed.
