# Supplementary material for: Efficacy of Cipargamin (KAE609) in a Randomized, Phase II Dose-Escalation Study in Adults in Sub-Saharan Africa With Uncomplicated Plasmodium falciparum Malaria
Source: Clin Infect Dis. 2021 Aug 19;74(10):1831–9. doi: 10.1093/cid/ciab716 (PMC9155642; doi:10.1093/cid/ciab716)
Supplement: ciab716_suppl_Supplementary_Materials [file ciab716_suppl_supplementary_materials.docx]

**Supplementary appendix**

This appendix has been provided by the authors to give readers additional information about their work.

Supplement to:
Schmitt, EK, Ndayisaba, G, Yeka, A, et al. Efficacy of cipargamin (KAE609) in a randomized, Phase II dose-escalation study in adults in sub-Saharan Africa with uncomplicated *Plasmodium falciparum* malaria.

**Contents**

The CKAE609A2202 study group 2

Full inclusion and exclusion criteria 4

Pfatp4 sequencing method...................................................................................................................6
Bioanalytical method used for determination of Cipargamin in patients.............................................7

Supplementary Figure 1. Patient Disposition.......................................................................................8

Supplementary Table 1. Decision criteria for cipargamin dose escalation based on LFT results 9

Supplementary Table 2. Proportion of patients with ETF, LCF or LPF (Full analysis set)................10

Supplementary Table 3. Summary of baseline demographics and disease characteristics
(Randomised set) .....11

Supplementary Table 4. Proportion of subjects with PCR-uncorrected ACPR at Day 15 and Day 29 (Full analysis set) 13

Supplementary Table 5. Summary statistics of cipargamin pharmacokinetic parameters (Pharmacokinetic analysis set) .......14

Supplementary Table 6. Summary of Pfatp4 mutations....................................................................15

**The CKAE609A2202 study group**

In addition to the authors, the following study group members were all closely involved with the implementation, conduct and oversight of the clinical trial. Study centers are listed in alphabetical order.

**Center for Family Health Research, Kigali, Rwanda (author: Etienne Karita)**

Julien Nyombayire

Rosine Ingabire

Jean Bizimana

**Centre de Recherches Médicales en Lambaréné, Lambaréné, Gabon (author: Martin P. Grobusch)**

Ghyslain Mombo-Ngoma

Rella Zoleko Manego

Peter Kremsner

**Infectious Diseases Research Collaboration, Busia, Uganda (author: Adoke Yeka)**

Afizi Kibuuka

Kenneth Kalamba

Juliet Imokol

Annet Nalwoga

**Joint Clinical Research Centre, Kampala, Uganda (author: Henry Mugerwa)**

Esther Kabaswaga

Margret Ndagire

Peter Erimu

Dridah Nakiboneka

**Kabwohe Clinical Research Center, Sheema, Uganda (author: Stephen Asiimwe)**

Anacret Byamukama

Edna Brenda Tindimwebwa

Edgar Nduho Ngarame

John Paul Ninkusiima

**Kintampo Health Research Centre, Kintampo North Municipality, Ghana (author: Kwaku Poku Asante)**

Seyram Kaali

Prince Agyapong Darko

Samuel Harrison

Elvis Wilson

**Malaria Research and Training Center, Faculty of Pharmacy, University of Science, Techniques and Technologies of Bamako, Bamako, Mali (author: Bakary Fofana)**

Mohamed Lamine Alhousseini

Amadou Bamadio

Souleymane Dama

**Navrongo Health Research Centre, Ghana Health Service, Navrongo, Ghana (author: Abraham Oduro)**

Thomas Anyorigiya

Michael Kaburise

Jonas Kulariba

Mathilda Tsifodze

**Rinda Ubuzima, Kigali, Rwanda (author: Gilles Ndayisaba)**

Jean Claude Ndagijimana

Marie Michelle Umulisa

Rosette Busasa

Lambert Mwambarangwe

**University Clinical Research Center, University of Sciences, Techniques and Technology of Bamako*,* Bamako, Mali (author: Seydou Doumbia)**

Mahamadou Diakite

Sory Ibrahima Diawara

Drissa Konate

Mariam Coulibaly

**Full inclusion and exclusion criteria**

**Inclusion criteria**

Subjects eligible for inclusion in this study fulfilled all of the following criteria:

**Demography**

1. Male and female subjects ≥ 18 years with a body weight ≥ 45 kg

**Disease specific**

1. Microscopic confirmation of acute uncomplicated *Plasmodium falciparum* using by Giemsa-stained thick film
2. *Plasmodium falciparum* parasitemia of 500 to 50 000 parasites/µL

**Health status**

1. Axillary temperature ≥ 37.5ºC or oral/tympanic/rectal temperature ≥ 38.0ºC; or history of fever during the previous 24 hours
2. Negative pregnancy test for subjects of childbearing potential

**Regulations**

1. Written informed consent must have been obtained before any study assessment was performed. If the subject was unable to write, then a witnessed consent according to local ethical standards was permitted
2. The subject was able to understand and comply with protocol requirements, instructions and protocol-stated restrictions and was likely to complete the study as planned
3. Living within reachable distance to the trial site to enable attendance for follow-up visits

**Exclusion criteria**

Subjects fulfilling any of the following criteria were not eligible for inclusion in this study.

**Medical history and clinical status**

1. Mixed *Plasmodiun* infections
2. Signs and symptoms of severe malaria according to World Health Organization (WHO) 2015 criteria
3. Active tuberculosis, or history of taking anti-tuberculosis medications within 12 months prior to screening
4. History of, or current alcohol misuse/abuse defined as five or more drinks on the same occasion on each of 5 or more days in the past 30 days
5. Known liver abnormalities, liver cirrhosis (compensated or decompensated), known active or history of hepatitis B or C (testing not required), known gallbladder or bile duct disease, acute or chronic pancreatitis
6. Clinical or laboratory evidence of any of the following:
   - AST/ALT > 1.5 x ULN, regardless of the level of total bilirubin
   - AST/ALT > 1.0 and ≤ 1.5 x ULN and total bilirubin > ULN
   - Total bilirubin > 2 x ULN, regardless of the level of AST/ALT
7. History of photodermatitis/increased sensitivity to sun
8. Known disturbances of electrolyte balance, e.g. hypokalemia, hypocalcemia or hypomagnesemia
9. Moderate to severe anemia (Hemoglobin level < 8 g/dL)
10. Any confirmed or suspected immunosuppressive or immunodeficient condition, including human immunodeficiency virus (HIV) infection
11. Severe malnutrition (Body Mass Index (BMI) < 16.0)
12. Severe vomiting, defined as more than 3 times in the 24 hours prior to inclusion in the study or severe diarrhea defined as more than 3 times watery stools per day
13. Pregnant or nursing (lactating) women
14. Sexually active subjects not willing to practice effective contraception
15. Women of child-bearing potential, defined as all women physiologically capable of becoming pregnant, unless they were using highly effective methods of contraception during dosing and for 30 days after administration of KAE609
16. Sexually active males were required to use a condom during intercourse while taking drug and for at least 30 days after stopping the study medication and should not father a child in this period. A condom was required to be used also by vasectomized men in order to prevent delivery of the drug via seminal fluid
17. Subjects with a family history of congenital prolongation of the QTc interval or sudden death or with any other clinical condition known to prolong the QTc interval such as subjects with a history of symptomatic cardiac arrhythmias, with clinically relevant bradycardia or with severe cardiac disease
18. Subjects who were taking drugs that are known to prolong the QTc interval
19. Resting QTcF > 450 ms (males), QTcF > 460 ms (females) at screening
20. Any surgical or medical condition which might have significantly altered the absorption, distribution, metabolism, or excretion of drugs, or which might have jeopardized the subject in case of participation in the study. The investigator should make this determination in consideration of the subject’s medical history, clinical and/or laboratory results.
21. History of malignancy of any organ system (other than localized basal cell carcinoma of the skin or *in situ* cervical cancer), treated or untreated, within the past 5 years, regardless of whether there was evidence of local recurrence or metastases
22. Known chronic underlying disease such as sickle cell disease, and severe cardiac impairment.
23. Subjects with serum creatinine ≥ 2 X ULN in the absence of dehydration. In case of dehydration, subjects with serum creatinine ≥2 X ULN after oral or parenteral rehydration.
24. Inability to tolerate oral medication, to drink.
25. Subjects taking any drug which is metabolized by the cytochrome (CYP) 2D6 enzyme (e.g., flecainide, metoprolol, imipramine, amitriptyline, clomipramine).
26. Subjects taking drugs that are strong inducers of CYP3A4 such as rifampicin, carbamazepine, phenytoin, St. John’s wort (Hypericum perforatum)
27. Known hypersensitivity to any of the agents used in the study

**Interfering substances**

1. Subjects with prior antimalarial therapy or antibiotics with antimalarial activity within less than five (5) plasma half-lives (or within 4 weeks of screening if half-life is unknown) Subjects with prior herbal medication within one week of screening
2. Subjects with prior herbal medication within one week of screening
3. Use of other investigational drugs within 5 half-lives of enrollment, or within 30 days or until the expected pharmacodynamic (PD) effect has returned to baseline, whichever was longer
4. Subjects who were taking medications prohibited by the protocol
5. Previous participation in any malaria vaccine study or received malaria vaccine in any other circumstance

**Pfatp4 sequencing and analysis**

Two Pfatp4 gene fragments were amplified by nested PCR. The first amplicon was 1071bp and used the forward primer 5’- tctattgtaaaagtagaagaatcacc-3’ and the reverse primer 5’-tccttcagttaatgtaccggt-3’ for the outer PCR. The inner primer pair were 5’-aagatgaagtggtacctgtatg -3’ and 5’-tcctaaagtttcaacagctggt -3’ yielding a 854bp amplicon (nucleotide position 476-1307). Both inner primers were also used for sequencing.

The second Pfatp4 fragment was amplified with 5’-agagcacaaccggaagataa -3’ and 5’-ttttaccacatgtacattttaaaca -3’ yielding an amplicon of 1324bp. The nested PCR was conducted with 5’-ctatgacaggagatggagtt-3’ and 5’-attttcttctatatataacctttgg -3’ covering nucleotides 2582 to 3760. The inner primers were again used for sequencing.

Sequences were compared to the reference genome 3D7. Baseline sequences from all patients in the study were analyzed for the presence of the following mutations.

| **Mutation** |
| --- |
| Q172H |
| Q172K |
| A184S |
| A187V |
| I203L |
| I203M |
| V204L |
| A211T |
| G223R |
| I263V |
| S312P |
| L350V |
| A353Q |
| G358S |
| S374R |
| I379N |
| I398F |
| V400A |
| V414D |
| T416N |
| T418N |
| A421E |
| E895K |
| L938I |
| P966A |
| A967G |
| P990R |
| A1158V |
| A1207V |
| D1247Y |

For patients with recrudescence after cipargamin treatment the baseline and treatment failure sample were analyzed in more detail to detect all treatment emerging mutations and possible mixed infections. Results are reported in supplementary table 6.

**Bioanalytical method used for determination of Cipargamin in patients**

For bioanalysis, plasma sample preparation consisted of protein precipitation, evaporation of the supernatants, and analysis of the reconstituted sample using liquid chromatography-tandem mass spectrometry (LC-MS/MS) with electrospray ionization (ESI) technique.

KAE609[M + 6] was used as the internal standard. The lower and upper limits of quantification were 1.00 ng/mL and 1,000 ng/mL, respectively, using 50 µL of plasma. Cipargamin is stable in human plasma for at least 24 h at room temperature, after 3 freeze-thaw cycles, and at -75°C±10°C for at least 395 days. The coefficient of variation (CV%; precision) and percent bias (accuracy) for the multiple runs during the period of analysis for low (3 ng/mL), mid (300) and high (750 ng/mL) quality control samples were 11.3 and -1.00, 8.14 and 2.00, and 9.08 and -6.27, respectively.

**Supplementary Figure 1. Patient disposition**


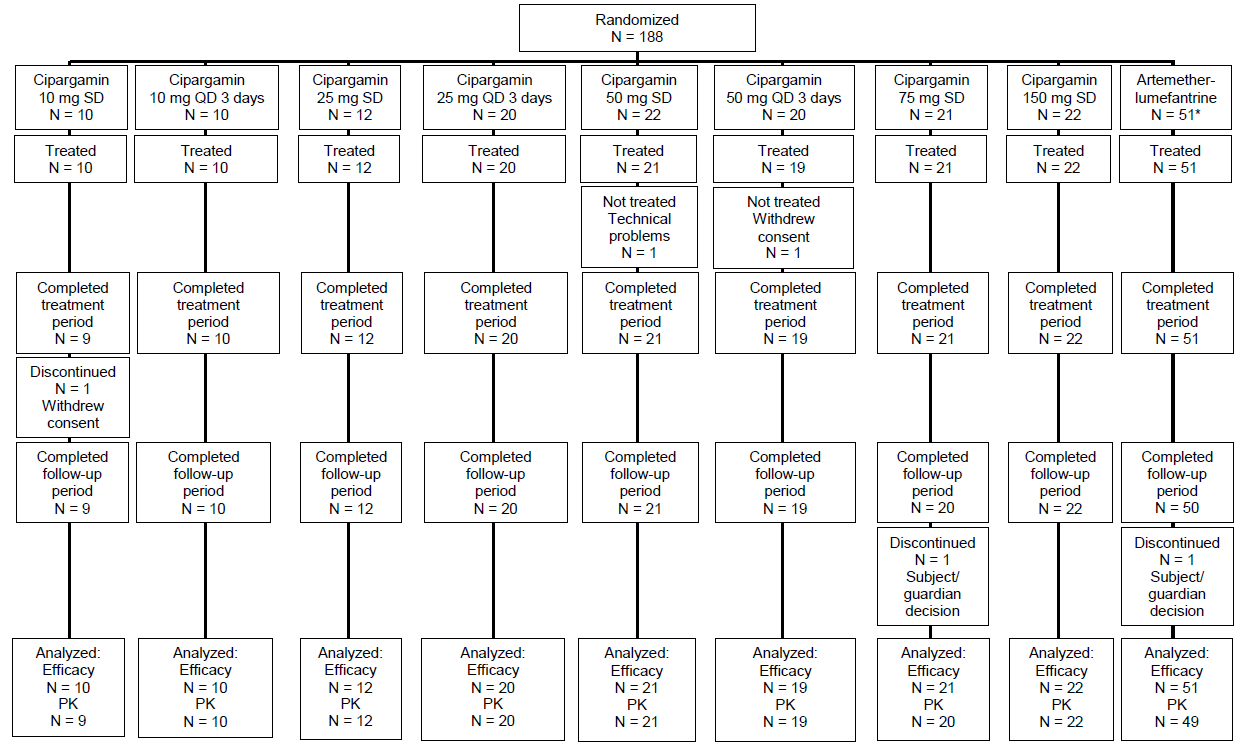


*Pooled artemether-lumefantrine groups from all cohorts

**Supplementary Table 1. Decision criteria for cipargamin dose escalation based on LFT results**

| **LFT parameter** | **Baseline** | **Maximum post baseline value** | **Decision to escalate to next cohort** |
| --- | --- | --- | --- |
| ALT/AST | Within ULN | < 2 times ULN (from **Day 1 to Day 15**) | Escalate to next cohort after notification to safety review committee, after all the patients in the cohort have been followed for at least 14 days post treatment (Study Day 15). |
|  | > 1 to ≤ 1.5 ULN | < 2 times baseline (from **Day 1 to Day 15**) |  |
| ALT/AST | Within ULN | ≥ 2 to < 3 times ULN (from **Day 1 to Day 15**) | Escalate to next cohort based on review of liver safety (and any other relevant data) by safety review committee, after all the patients in the cohort have been followed for at least 28 days post treatment (**Study Day 29**) |
|  | > 1 to ≤ 1.5 ULN | ≥ 2 to < 3 times baseline (from **Day 1 to Day 15**) |  |
| ALT/AST | Any baseline value | ≥ 2 Grade (CTCAE grades) increase from baseline (**at any time point during the study**) in: 2 patients in a 10-patient cohort (Cohorts 1 and 2)  or  3 patients in a 20-patient cohort (Cohorts 3, 4, 5, and 6) | Suspend recruitment and initiate review of liver safety (and any other relevant data) by safety review committee.  Any further progression of the study is based on the decision by the safety review committee |
| ALT: alanine aminotransferase, AST: aspartate aminotransferase, ULN: upper limit of the normal range | | | |

**Supplementary Table 2. Proportion of patients with ETF, LCF or LPF (Full analysis set)**

| **Endpoint** | **Treatment group** | **n/M (%)** | **Exact 95% CI (%)** |
| --- | --- | --- | --- |
| Early treatment failure (ETF) | KAE609 10 mg SD (N=10) | 0/9 (0.0) | (0.00,33.63) |
|  | KAE609 10 mg QD 3 days (N=10) | 0/10 (0.0) | (0.00,30.85) |
|  | KAE609 25 mg SD (N=12) | 0/12 (0.0) | (0.00,26.46) |
|  | KAE609 25 mg QD 3 days (N=20) | 0/20 (0.0) | (0.00,16.84) |
|  | KAE609 50 mg SD (N=21) | 0/21 (0.0) | (0.00,16.11) |
|  | KAE609 50 mg QD 3 days (N=19) | 0/18 (0.0) | (0.00,18.53) |
|  | KAE609 75 mg SD (N=21) | 0/21 (0.0) | (0.00,16.11) |
|  | KAE609 150 mg SD (N=22) | 0/22 (0.0) | (0.00,15.44) |
|  | Pooled Coartem (N=51) | 1/48 (2.1) | (0.05,11.07) |
| Late clinical failure (LCF) | KAE609 10 mg SD (N=10) | 0/9 (0.0) | (0.00,33.63) |
|  | KAE609 10 mg QD 3 days (N=10) | 0/10 (0.0) | (0.00,30.85) |
|  | KAE609 25 mg SD (N=12) | 1/12 (8.3) | (0.21,38.48) |
|  | KAE609 25 mg QD 3 days (N=20) | 0/20 (0.0) | (0.00,16.84) |
|  | KAE609 50 mg SD (N=21) | 0/21 (0.0) | (0.00,16.11) |
|  | KAE609 50 mg QD 3 days (N=19) | 1/19 (5.3) | (0.13,26.03) |
|  | KAE609 75 mg SD (N=21) | 2/20 (10.0) | (1.23,31.70) |
|  | KAE609 150 mg SD (N=22) | 2/22 (9.1) | (1.12,29.16) |
|  | Pooled Coartem (N=51) | 0/49 (0.0) | (0.00,7.25) |
| Late parasitological failure (LPF) | KAE609 10 mg SD (N=10) | 1/9 (11.1) | (0.28,48.25) |
|  | KAE609 10 mg QD 3 days (N=10) | 1/10 (10.0) | (0.25,44.50) |
|  | KAE609 25 mg SD (N=12) | 3/11 (27.3) | (6.02,60.97) |
|  | KAE609 25 mg QD 3 days (N=20) | 4/20 (20.0) | (5.73,43.66) |
|  | KAE609 50 mg SD (N=21) | 4/21 (19.0) | (5.45,41.91) |
|  | KAE609 50 mg QD 3 days (N=19) | 5/18 (27.8) | (9.69,53.48) |
|  | KAE609 75 mg SD (N=21) | 3/18 (16.7) | (3.58,41.42) |
|  | KAE609 150 mg SD (N=22) | 7/20 (35.0) | (15.39,59.22) |
|  | Pooled Coartem (N=51) | 2/49 (4.1) | (0.50,13.98) |
| ETF: defined as: 1. Clinical decline or lack of improvement (per Investigator) at 24 hours after first dose of treatment. 2. Development of any clinical complications (described in the WHO definition of complicated/severe malaria [13] in the presence of parasitemia within 72 hours of the first dose of treatment 3. Parasitemia > 75 000/μL at or after 12 hours post-dose 4. Parasitemia > baseline with or without fever at 36 hours after first dose of treatment 5. Any parasitemia based on microscopy with fever, 48 hours post-dose 6. Parasitemia > 100/ μL based on microscopy with or without fever, 72 hours post-dose The ETF definition in this study is stricter than the WHO definition [14].  LTF: defined as: Development of danger signs or severe malaria on any day between Study Day 5 and Day 29 in the presence of parasitemia without previously meeting any of the criteria of ETF. Presence of parasitemia and axillary temperature ≥37.5ºC on any day between Study Day 5 and Day 29 without previously meeting any of the criteria of ETF.  LCF: defined as: Presence of parasitemia on any day between Study Day 5 and Day 29 and axillary temperature <37.5ºC without previously meeting any of the criteria of ETF or LCF. The uncorrected parasite counts are used for calculations. Confidence interval is based on the Clopper-Pearson method. M = For ETF, no. of subjects with ETF or with assessment of parasitaemia at Day 4; for LCF, no. of subjects with LCF or with assessment of parasitaemia at Day 29 and not ETF; for LPF, no. of subjects with LPF or with assessment of parasitaemia at Day 29 and not ETF/LCF. | | | |

**Supplementary Table 3. Summary of baseline demographics and disease characteristics (Randomised set)**

| **Characteristic** | **Cipargamin 10 mg single dose N=10** | **Cipargamin 10 mg QD/3 days N=10** | **Cipargamin 25 mg single dose N=12** | **Cipargamin 25 mg QD/3 days N=20** | **Cipargamin 50 mg single dose N=21** | **Cipargamin 50 mg QD/3 days N=19** | **Cipargamin 75 mg single dose N=21** | **Cipargamin 150 mg single dose N=22** | **Pooled Artemether-lumefantrine N=51** |
| --- | --- | --- | --- | --- | --- | --- | --- | --- | --- |
| **Age (years)** |  |  |  |  |  |  |  |  |  |
| Mean (SD) | 31.5 (10.48) | 35.4 (13.25) | 33.0 (14.96) | 31.9 (10.67) | 33.9 (12.56) | 26.4 (7.18) | 28.2 (10.25) | 30.6 (12.57) | 26.2 (9.07) |
| Median | 33.5 | 35.5 | 33.5 | 30.5 | 28.0 | 25.0 | 26.0 | 24.0 | 23.0 |
| Min-Max | 18-48 | 18-55 | 18-60 | 18-50 | 18-58 | 18-46 | 18-61 | 18-58 | 18-53 |
| **Age group - n (%)** |  |  |  |  |  |  |  |  |  |
| < 65 years | 10 (100) | 10 (100) | 12 (100) | 20 (100) | 22 (100) | 20 (100) | 21 (100) | 22 (100) | 51 (100) |
| **Sex - n (%)** |  |  |  |  |  |  |  |  |  |
| Male | 8 (80.0) | 4 (40.0) | 4 (33.3) | 9 (45.0) | 13 (59.1) | 12 (60.0) | 16 (76.2) | 16 (72.7) | 33 (64.7) |
| Female | 2 (20.0) | 6 (60.0) | 8 (66.7) | 11 (55.0) | 9 (40.9) | 8 (40.0) | 5 (23.8) | 6 (27.3) | 18 (35.3) |
| **BMI (kg/m2)** |  |  |  |  |  |  |  |  |  |
| Mean (SD) | 20.22 (2.815) | 23.55 (4.263) | 23.86 (4.436) | 22.57 (3.743) | 23.71 (4.323) | 21.81 (2.718) | 21.21 (2.133) | 22.35 (2.984) | 22.30 (3.503) |
| Median | 19.64 | 23.14 | 22.98 | 21.34 | 22.15 | 21.32 | 21.14 | 22.01 | 21.96 |
| Min-Max | 17.1-25.5 | 18.3-30.8 | 17.8-32.1 | 17.4-32.5 | 17.8-35.8 | 18.3-29.4 | 17.8-26.1 | 17.2-30.9 | 17.4-36.8 |
| **BMI - n (%)** |  |  |  |  |  |  |  |  |  |
| < 16 kg/m2 | 0 | 0 | 0 | 0 | 0 | 0 | 0 | 0 | 0 |
| 16 - 25 kg/m2 | 9 (90.0) | 7 (70.0) | 8 (66.7) | 16 (80.0) | 15 (68.2) | 18 (90.0) | 19 (90.5) | 19 (86.4) | 45 (88.2) |
| > 25 kg/m2 | 1 (10.0) | 3 (30.0) | 4 (33.3) | 4 (20.0) | 7 (31.8) | 2 (10.0) | 2 (9.5) | 3 (13.6) | 6 (11.8) |
| **Body temperature (°C)** | |  |  |  |  |  |  |  |  |
| n | 10 | 10 | 12 | 20 | 22 | 20 | 21 | 22 | 51 |
| Mean (SD) | 37.30 (0.872) | 36.98 (0.973) | 36.41 (0.571) | 37.03 (0.585) | 36.88 (0.874) | 36.76 (1.019) | 37.00 (1.133) | 36.80 (1.039) | 37.18 (1.045) |
| Median | 37.35 | 36.60 | 36.25 | 37.00 | 36.60 | 36.60 | 36.80 | 36.45 | 37.00 |
| Min-Max | 36.2-39.3 | 36.1-39.0 | 35.6-37.4 | 36.3-38.6 | 35.9-39.3 | 35.2-38.6 | 35.2-39.9 | 35.0-39.2 | 35.6-39.7 |
| **Body temperature (axillary) category (°C) - n (%)** | | | |  |  |  |  |  |  |
| < 37.5 | 1 (10.0) | 7 (70.0) | 11 (91.7) | 11 (55.0) | 11 (50.0) | 15 (75.0) | 15 (71.4) | 17 (77.3) | 27 (52.9) |
| 37.5 - < 39 | 0 | 1 (10.0) | 0 | 1 (5.0) | 2 (9.1) | 5 (25.0) | 4 (19.0) | 4 (18.2) | 9 (17.6) |
| ≥ 39 | 0 | 1 (10.0) | 0 | 0 | 1 (4.5) | 0 | 2 (9.5) | 1 (4.5) | 5 (9.8) |

| **Characteristic** | **Cipargamin 10 mg single dose N=10** | **Cipargamin 10 mg QD/3 days N=10** | **Cipargamin 25 mg single dose N=12** | **Cipargamin 25 mg QD/3 days N=20** | **Cipargamin 50 mg single dose N=21** | **Cipargamin 50 mg QD/3 days N=19** | **Cipargamin 75 mg single dose N=21** | **Cipargamin 150 mg single dose N=22** | **Pooled Artemether-lumefantrine N=51** |
| --- | --- | --- | --- | --- | --- | --- | --- | --- | --- |
| ***P. falciparum* density (/**µ**L)** | |  |  |  |  |  |  |  |  |
| Mean | 3297.4 | 9883.5 | 6905.8 | 8736.6 | 8399.7 | 16992.8 | 13634.3 | 14820.8 | 14753.9 |
| (SD) | (4263.87) | (14537.19) | (10520.48) | (8817.24) | (7247.61) | (9758.97) | (12698.30) | (12633.46) | (12129.56) |
| Median | 850.0 | 1736.0 | 2406.0 | 6430.0 | 5641.5 | 15696.5 | 8190.0 | 9455.5 | 12800.0 |
| Min-Max | 502-11105 | 617-46600 | 673-37681 | 725-34580 | 639-29251 | 1654-32471 | 568-39663 | 1163-42955 | 608-47363 |
| ***P. falciparum* density (/**µ**L) categories - n (%)** | | | |  |  |  |  |  |  |
| < 500 | 0 | 0 | 0 | 0 | 0 | 0 | 0 | 0 | 0 |
| 500 - < 2,000 | 7 (70.0) | 6 (60.0) | 6 (50.0) | 4 (20.0) | 5 (22.7) | 1 (5.0) | 1 (4.8) | 1 (4.5) | 13 (25.5) |
| 2,000 - < 5,000 | 0 | 0 | 1 (8.3) | 5 (25.0) | 5 (22.7) | 2 (10.0) | 3 (14.3) | 5 (22.7) | 3 (5.9) |
| 5,000 - < 15,000 | 3 (30.0) | 2 (20.0) | 4 (33.3) | 7 (35.0) | 8 (36.4) | 5 (25.0) | 10 (47.6) | 8 (36.4) | 12 (23.5) |
| 15,000 - < 50,000 | 0 | 2 (20.0) | 1 (8.3) | 4 (20.0) | 4 (18.2) | 12 (60.0) | 7 (33.3) | 8 (36.4) | 23 (45.1) |
| ≥ 50,000 | 0 | 0 | 0 | 0 | 0 | 0 | 0 | 0 | 0 |

**Supplementary Table 4. Proportion of subjects with PCR-uncorrected ACPR at Day 15 and Day 29 (Full analysis set)**

| **Treatment group** | **Time point** | **n (%) with ACPR** | **Exact 95% Confidence Interval (%)** |
| --- | --- | --- | --- |
| KAE609 10 mg SD (N=10) | Day 15 | 9 (90.0) | (55.50, 99.75) |
|  | Day 29 | 8 (80.0) | (44.39, 97.48) |
| KAE609 10 mg QD 3 days (N=10) | Day 15 | 9 (90.0) | (55.50, 99.75) |
|  | Day 29 | 9 (90.0) | (55.50, 99.75) |
| KAE609 25 mg SD (N=12) | Day 15 | 10 (83.3) | (51.59, 97.91) |
|  | Day 29 | 8 (66.7) | (34.89, 90.08) |
| KAE609 25 mg QD 3 days (N=20) | Day 15 | 19 (95.0) | (75.13, 99.87) |
|  | Day 29 | 16 (80.0) | (56.34, 94.27) |
| KAE609 50 mg SD (N=21) | Day 15 | 20 (95.2) | (76.18, 99.88) |
|  | Day 29 | 17 (81.0) | (58.09, 94.55) |
| KAE609 50 mg QD 3 days (N=19) | Day 15 | 16 (84.2) | (60.42, 96.62) |
|  | Day 29 | 13 (68.4) | (43.45, 87.42) |
| KAE609 75 mg SD (N=21) | Day 15 | 18 (85.7) | (63.66, 96.95) |
|  | Day 29 | 15 (71.4) | (47.82, 88.72) |
| KAE609 150 mg SD (N=22) | Day 15 | 17 (77.3) | (54.63, 92.18) |
|  | Day 29 | 13 (59.1) | (36.35, 79.29) |
| Pooled Coartem (N=51) | Day 15 | 49 (96.1) | (86.54, 99.52) |
|  | Day 29 | 47 (92.2) | (81.12, 97.82) |

Missing blood smear data at Day 15 visit and thereafter are not considered as responder for the visit unless there is a later blood smear test indicating no parasitemia.

Confidence interval is based on Clopper-Pearson method.

**Supplementary Table 5. Summary statistics of cipargamin pharmacokinetic parameters by treatment**

|  | **Cipargamin dose/regimen** | | | | | | | |
| --- | --- | --- | --- | --- | --- | --- | --- | --- |
| **Parameter** | **10mg SD N=9** | **10mg QD 3 days N=10** | **25mg SD N=12** | **25mg QD 3 days N=20** | **50mg SD N=21** | **50mg QD 3 days N=19** | **75mg SD N=20** | **150mg SD N=22** |
| C_max_ (ng/mL) |  |  |  |  |  |  |  |  |
| Day 1 | 190 +/- 74.8 | 206 +/- 110 | 408 +/- 159 | 546 +/- 214 | 810 +/- 256 | 870 +/- 256 | 1350 +/- 442 | 2450 +/- 691 |
|  | [39.3] | [53.1] | [39.1] | [39.2] | [31.5] | [29.4] | [32.7] | [28.3] |
| Day 3 |  | 250 +/- 97.0 |  | 678 +/- 178 |  | 1260 +/- 378 |  |  |
|  |  | [38.8] |  | [26.2] |  | [30.0] |  |  |
| T_max_ (h) |  |  |  |  |  |  |  |  |
| Day 1 | 4.00 (1.02-12.1) | 3.92 (1.03-6.17) | 4.01 (2.00-8.33) | 4.25 (2.00-23.8) | 4.12 (1.97-10.0) | 4.12 (2.00-6.13) | 6.01 (3.97-12.3) | 8.07 (1.00-24.1) |
| Day 3 |  | 52.7 (50.0-60.6) |  | 52.1 (49.9-59.6) |  | 52.0 (50.0-60.5) |  |  |
| AUC_last_ (h*µg/mL) | |  |  |  |  |  |  |  |
| Day 1 | 6.60 +/- 3.35 | 3.16 +/- 1.58 | 13.8 +/- 5.63 | 8.91 +/- 3.46 | 29.4 +/- 9.64 | 14.8 +/- 5.30 | 59.5 +/- 25.4 | 113 +/- 32.4 |
|  | [50.8] | [50.0] | [40.7] | [38.8] | [32.8] | [35.9] | [42.6] | [28.8] |
| Day 3 |  | 11.0 +/- 5.44 |  | 30.2 +/- 11.8 |  | 55.7 +/- 19.1 |  |  |
|  |  | [49.7] |  | [39.0] |  | [34.4] |  |  |
| AUC_inf_ (h*µg/mL) | |  |  |  |  |  |  |  |
| Day 1 | 6.63 +/- 3.80 |  | 15.1 +/- 5.66 |  | 30.6 +/- 9.62 |  | 62.5 +/- 24.1 | 117 +/- 35.2 |
|  | [57.3] |  | [37.6] |  | [31.5] |  | [38.6] | [30.0] |
| Day 3 |  | 12.2 +/- 6.44 |  | 30.7 +/- 12.4 |  | 50.6 +/- 12.7 |  |  |
|  |  | [52.6] |  | [40.3] |  | [25.2] |  |  |
| AUC_0-24_ (h*µg/mL) | |  |  |  |  |  |  |  |
| Day 1 | 3.05 +/- 1.45 | 2.75 +/- 0.957 | 5.67 +/- 2.46 | 9.10 +/- 3.68 | 12.3 +/- 4.35 | 16.1 +/- 4.06 | 22.9 +/- 7.77 | 41.7 +/- 10.7 |
|  | [47.7] | [34.8] | [43.4] | [40.5] | [35.3] | [25.2] | [33.9] | [25.6] |
| Day 3 |  | 4.18 +/- 1.87 |  | 11.3 +/- 3.18 |  | 22.6 +/- 6.85 |  |  |
|  |  | [44.8] |  | [28.1] |  | [30.4] |  |  |
| T_1/2_ (h) |  |  |  |  |  |  |  |  |
| Day 1 | 24.4 +/- 8.70 | 18.5 +/- 6.24 | 35.1 +/- 13.9 | 17.4 +/- 3.27 | 31.5 +/- 17.4 | 32.8 +/- 5.05 | 25.3 +/- 8.94 | 29.9 +/- 12.5 |
|  | [35.6] | [33.8] | [39.5] | [18.8] | [55.3] | [15.4] | [35.3] | [41.7] |
| Day 3 T_1/2_ (h) |  | 32.4 +/- 14.8 |  | 30.1 +/- 14.0 |  | 29.9 +/- 22.0 |  |  |
|  |  | [45.7] |  | [46.3] |  | [73.6] |  |  |
| Blood samples for PK analysis were obtained at 1, 2, 4, 6, 8, 12, 24, 72 and 168 hours after dosing in patients receiving single doses of cipargamin, and at 1, 2, 4, 6, 24, 48, 50, 52, 56, 60, 72, and 168 hours after first dose in patients receiving multiple cipargamin doses. All values are mean +/- SD [CV%] except for Tmax which is median (range). Coefficient of variation (in %) is given in square brackets. | | | | | | | | |

**Supplementary Table 6. Summary of treatment emerging mutations in Pfatp4 gene from patients with recrudescence, which were not present at baseline**

| **Mutation at recrudescence** | **Number of patients** |
| --- | --- |
| G358S clean | 17 |
|  |  |
| G358S/A mixed | 2 |
|  |  |
| G358S and WT mixed | 2 |
| G358S and G359A mixed | 1 |
|  |  |
| G359A clean | 1 |
|  |  |
| L354V | 1 |
|  |  |
| L181F | 1 |

G223S (14%) and Q1081K (76%) and G1128R (79%) were identified in baseline samples as well as recrudescent parasites but no correlation with treatment failure could be established.
